# State-Dependent Modulation of Neurotransmitter Systems in Epilepsy: A Mechanistic Framework for Seizure Dynamics and Biomarker Variability

**DOI:** 10.3390/biology15110850

**Published:** 2026-05-29

**Authors:** Ekaterina Andreevna Narodova

**Affiliations:** Department of Neurology, Prof. V.F. Voyno-Yasenetsky Krasnoyarsk State Medical University, 660022 Krasnoyarsk, Russia; katya_n2001@mail.ru

**Keywords:** epilepsy, neurotransmitter systems, state-dependent modulation, network excitability, seizure dynamics, biomarkers, synaptic plasticity, ion channels

## Abstract

Epilepsy is increasingly conceptualized as a disorder of dynamic network instability rather than a static imbalance between excitation and inhibition. However, substantial variability in seizure occurrence, clinical expression, and treatment response remains insufficiently explained by existing models. This narrative review examines how neurotransmitter systems contribute to seizure dynamics within a state-dependent framework, in which factors such as sleep–wake cycles, stress, inflammation, and metabolic conditions modulate network excitability. The review identified four key findings: neurotransmitter function in epilepsy is state-dependent rather than fixed; multiple physiological state modifiers shape seizure susceptibility; seizure termination is an active state-sensitive process; and biomarker performance depends on the prevailing brain state. Evidence from experimental and clinical studies indicates that neurotransmitter function is context-sensitive and interacts with molecular pathways, including ion channel function, synaptic plasticity, and neuromodulatory signaling. These interactions influence key stages of seizure dynamics, including initiation, propagation, and termination, and may differ across etiological categories of epilepsy. This perspective also helps explain the limited performance of static biomarkers, as they do not capture temporal variability in network states. Instead, state-sensitive markers and context-aware interpretations of electrophysiological and clinical data may provide more informative insights. Overall, integrating neurotransmitter mechanisms with dynamic brain states offers a more precise perspective on seizure variability and may support the development of individualized, state-aware approaches to epilepsy management.

## Simple Summary

Epilepsy is a neurological condition characterized by recurrent seizures, but patients often show substantial variability in when seizures occur, how they evolve, and how they respond to treatment. Traditional explanations usually focus on fixed abnormalities in brain chemistry, particularly imbalances between excitatory and inhibitory neurotransmitters. However, these approaches do not fully explain why seizure susceptibility changes over time within the same individual. In this review, I propose that neurotransmitter function in epilepsy should be understood as state-dependent, meaning that it varies depending on factors such as sleep, stress, inflammation, and metabolic conditions. Rather than representing static defects, neurotransmitter alterations may reflect dynamic changes in brain network activity. This perspective, which I propose in this review, helps explain clinical variability and highlights why static biomarkers often fail to predict seizure risk or treatment response. By linking molecular processes with brain states and network dynamics, this approach may support the development of more individualized and context-sensitive approaches to epilepsy management. The present review extends beyond existing models of network instability by explicitly proposing that neurotransmitter function serves as a state-dependent variable—one whose direction and magnitude of effect are determined by concurrent physiological context rather than by baseline molecular properties alone. Unlike prior reviews that describe neurotransmitter alterations as fixed contributors to epileptogenesis, this model reframes them as dynamic readouts of brain state. This reframing has direct implications for biomarker interpretation: rather than seeking stable molecular signatures, it suggests that clinically informative markers may only emerge under specific state conditions. I also suggest that this aligns with emerging translational directions, including state-dependent neurostimulation and chronotherapy, where timing of intervention relative to brain state may critically determine efficacy.

## 1. Introduction

Epilepsy is a heterogeneous neurological disorder characterized by recurrent unprovoked seizures arising from abnormal neuronal activity. Despite advances in neurobiology, the mechanisms underlying seizure variability—both across patients and within the same individual over time—remain incompletely understood [1,2]. Traditional models have largely emphasized a relatively static imbalance between excitatory and inhibitory neurotransmission, often focusing on γ-aminobutyric acid (GABA) and glutamate systems [3,4].

However, clinical observations indicate that seizure susceptibility is not constant but fluctuates over time, influenced by factors such as sleep–wake cycles, stress, metabolic state, and inflammatory processes. These observations suggest that neurotransmitter systems operate within a dynamic physiological context rather than as fixed determinants of network behavior. In parallel, increasing evidence from experimental and clinical studies indicates that neuronal excitability emerges from the interaction of multiple levels of organization, including ion channel function, synaptic plasticity, and neuromodulatory signaling [2,5,6,7,8].

Importantly, epilepsy encompasses a wide range of etiological categories, including genetic, structural, developmental, metabolic, immune, infectious, and unknown causes [1,9]. Although seizures represent the defining clinical feature of epilepsy, the underlying mechanisms leading to network instability may differ substantially across these categories. At the same time, common downstream pathways—such as altered neurotransmitter signaling and impaired regulation of excitability—may contribute to seizure generation across diverse forms of epilepsy [10,11,12].

State-dependent modulation of neurotransmitter systems may differ substantially in pediatric and developmental epilepsies, where age-dependent circuit maturation and evolving chloride homeostasis may modify the functional expression of these mechanisms [1,9,10].

Table 1 summarizes these etiological categories and illustrates how selected refractory epilepsies, including Dravet syndrome, Lennox–Gastaut syndrome, West syndrome, and Rasmussen syndrome, may be interpreted within the proposed downstream neurotransmitter/network framework.

This raises a conceptual challenge: how to reconcile etiological heterogeneity with partially shared mechanisms of network dysfunction. One possible approach is to consider neurotransmitter dysregulation not as a static abnormality but as a context-dependent process that varies across physiological and pathological states. Within this framework, factors such as sleep, stress, and metabolic conditions are not merely external modifiers but integral components that shape network excitability and seizure dynamics [13,14,15,16,17].

In this narrative review, I examine neurotransmitter systems in epilepsy within a state-dependent framework, with particular attention to how dynamic modulation influences seizure initiation, propagation, and termination. These relationships are inherently non-linear and context-dependent: the dominant pathways and their functional consequences may differ across etiologies, brain states, and disease stages. The review further discusses the implications of this perspective for biomarker development, emphasizing the limitations of static approaches and the potential value of state-sensitive markers. By integrating molecular mechanisms with network-level dynamics and clinical variability, this review aims to provide a more structured and mechanistically grounded interpretation of seizure heterogeneity in epilepsy.

Importantly, the present framework extends existing models of network instability and seizure dynamics by explicitly incorporating state-dependent variability as a central organizing principle. While prior approaches have emphasized dynamical systems, excitation–inhibition balance, and large-scale network interactions, they have typically treated neurotransmitter function and biomarkers as relatively stable properties.

In contrast, this review proposes that neurotransmitter activity should be interpreted as a context-sensitive variable that dynamically reflects ongoing brain states. This shift has several implications. First, it provides a mechanistic basis for intra-individual variability in seizure susceptibility that is not fully captured by static models. Second, it offers a structured explanation for the limited predictive value of single-timepoint biomarkers, suggesting that their interpretability depends on the underlying physiological state. Third, it supports the concept of state-aware clinical strategies, in which monitoring and intervention may be optimized according to temporal fluctuations in network excitability.

In this sense, the proposed framework does not replace existing models but reinterprets them within a state-dependent context, emphasizing temporal variability as a key dimension of epileptic network behavior.

For example, while dynamical systems approaches describe seizure transitions in terms of attractor states, they do not explicitly address how neurotransmitter function and biomarker interpretation vary across physiological states. Thus, the added value of the present framework is to connect dynamical descriptions of seizure transitions with state-sensitive molecular and clinical variables, thereby linking network-level theory to measurable fluctuations in neurotransmitter function, biomarker performance, and therapeutic timing.

**Figure 1:**
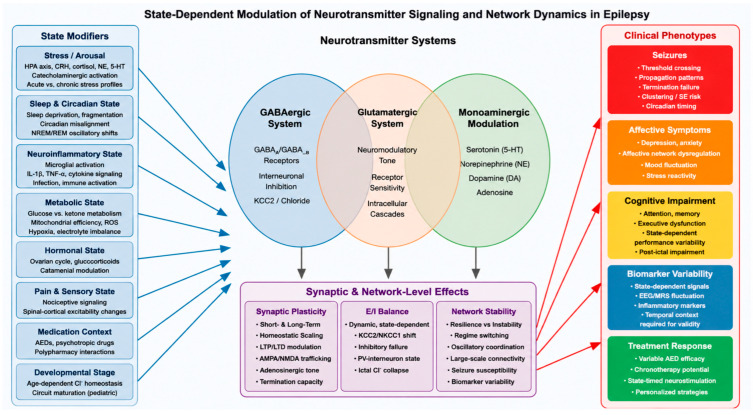
Conceptual model illustrating state-dependent modulation of neurotransmitter signaling and network dynamics in epilepsy. Multiple physiological state modifiers—including stress and arousal, sleep–wake cycles, neuroinflammatory activity, metabolic state, hormonal context, pain and sensory input, hypoxia and ionic imbalance, medication background, and developmental stage—act on GABAergic, glutamatergic, and monoaminergic systems, shaping excitation–inhibition balance, synaptic plasticity, and network stability. Molecular mechanisms include KCC2-dependent chloride homeostasis, AMPA/NMDA receptor trafficking, interneuronal function, and adenosinergic tone. Depending on the prevailing network regime, these interactions result in context-dependent expression of seizures (including altered termination dynamics), affective and cognitive symptoms, biomarker variability, and variable treatment response. Arrows indicate probabilistic and context-dependent modulatory relationships rather than fixed or linear causality.

These interactions are summarized in Figure 1.

Importantly, the primary contribution of this review lies not in identifying novel molecular mechanisms, but in reorganizing existing evidence into a structured, state-dependent framework that explicitly links neurotransmitter regulation with dynamic brain states, seizure variability, and biomarker interpretation. By integrating molecular, network, and clinical perspectives, this approach provides a coherent and testable conceptual model that addresses limitations of existing static paradigms. In contrast to many existing models, which typically conceptualize neurotransmitter dysfunction as a relatively stable contributor to epileptogenesis, the present framework explicitly treats neurotransmitter function as a dynamic, state-dependent variable. While dynamical systems approaches describe seizure transitions at the network level, they do not directly address how molecular and synaptic processes vary across physiological states or how this variability affects biomarker performance and clinical decision-making. By integrating these levels, the proposed model provides a more explicit link between molecular mechanisms, network dynamics, and clinically observable variability.

## 2. Materials and Methods

### 2.1. Study Design

This study was conducted as a narrative review aimed at synthesizing current evidence on neurotransmitter dysregulation in epilepsy within a state-dependent framework. Given the conceptual and integrative nature of the topic, a narrative approach was considered more appropriate than a formal systematic review, allowing the inclusion of mechanistic, experimental, and clinical studies across multiple levels of analysis.

The SANRA framework was used as a reporting-oriented self-assessment tool rather than as a formal scoring instrument for included studies [18]. The six SANRA domains were used as guiding principles for structured self-assessment of the manuscript: (1) justification of the review’s importance; (2) statement of concrete aims; (3) description of the literature search; (4) quality of referencing; (5) scientific reasoning; and (6) appropriate presentation of data and results. Each criterion was reviewed iteratively, and areas identified as requiring improvement were addressed through revision of the manuscript. This approach is consistent with structured methodological frameworks for narrative reviews that emphasize transparency, thematic rigor, and conceptual consistency of the synthesis process [18,19]. No formal inter-rater reliability assessment was performed, which is an acknowledged limitation of single-author narrative reviews.

### 2.2. Literature Search Strategy

A structured literature search was performed to identify relevant studies addressing neurotransmitter systems, network excitability, and state-dependent modulation in epilepsy.

Electronic databases including PubMed, Scopus, and Web of Science were searched for articles published up to January 2026. The search strategy combined keywords related to epilepsy, neurotransmission, and brain states. The main search terms included:

- “Epilepsy”;
- “Seizure”;
- “Neurotransmitter”;
- “GABA”;
- “Glutamate”;
- “Network excitability”;
- “Brain state”;
- “Sleep”;
- “Stress”;
- “Inflammation”;
- “Metabolism”;
- “Neuromodulation”.

These terms were used alone and in combination using Boolean operators (AND, OR).

In addition to database searches, reference lists of relevant articles were manually screened to identify further pertinent studies.

### 2.3. Eligibility Criteria

Studies were considered eligible if they met the following criteria:

- Addressed epilepsy or seizure-related mechanisms;
- Investigated neurotransmitter systems or neural excitability;
- Examined factors influencing brain state (e.g., sleep, stress, inflammation, metabolic conditions);
- Included experimental, clinical, or translational data.

Both original research articles and review papers were included.

Studies not directly related to epilepsy or lacking relevance to neurotransmitter function or state-dependent mechanisms were excluded.

### 2.4. Study Selection and Synthesis

The selection of studies was guided by relevance to the conceptual framework rather than predefined quantitative criteria. Priority was given to:

- Studies providing mechanistic insights;
- Well-established experimental findings;
- Clinically relevant observations;
- Frequently cited or foundational publications in the field.

The evidence was synthesized qualitatively, with a focus on identifying converging mechanisms across different levels of analysis rather than performing a quantitative meta-analysis.

Given the narrative design of this review, the study selection was guided by conceptual relevance rather than formal systematic criteria. As a result, the potential for selection bias cannot be excluded. In addition, no formal quality assessment of included studies was performed. Furthermore, literature search and study selection were conducted by a single author without independent verification, which represents an additional source of potential bias. These limitations should be considered when interpreting the findings. Where available, conflicting or non-supportive findings were considered during interpretation, particularly when evidence differed between experimental models and clinical studies or when associations were inconsistent across epilepsy syndromes. Such discrepancies were not treated as contradictory to the framework, but as indicators of context dependence and heterogeneity in the relationship between neurotransmitter regulation, brain state, and seizure expression.

### 2.5. Narrative Synthesis and Thematic Organization

The narrative synthesis was organized using predefined data-extraction fields and conceptual mapping categories presented in Table S2.

These categories were developed through iterative thematic grouping of the included literature and were used to structure the interpretation of evidence across molecular, synaptic, network, clinical, and translational levels.

Each of the 123 included articles was assigned to one or more conceptual mapping categories according to its primary focus.

The number of articles assigned to the narrative synthesis differs from the total number of references in the manuscript, because several additional references were used to support methodological, definitional, or contextual statements rather than being included in the conceptual domain mapping.

The distribution of studies across conceptual mapping categories was relatively balanced, although molecular/synaptic and network-level domains were most frequently represented, while translational and biomarker-focused domains were comparatively less represented. Because several studies addressed more than one level of analysis, domain assignment was not mutually exclusive. Approximately half of the included sources contributed to the neurotransmitter systems and network-level domains, while state modifier evidence was distributed across the remaining sources; translational and biomarker-focused domains drew on a comparatively smaller subset of the literature.

Specifically, approximately 45–50 sources contributed primarily to the neurotransmitter systems and network-level domains (Section 3 and the network portions of Section 4); approximately 35–40 sources addressed state modifier evidence (sleep, stress, inflammation, metabolic, and related modifiers); approximately 20–25 sources informed the seizure termination domain (Section 5); and approximately 15–20 sources were mapped to biomarker-related evidence (Section 6). The remaining sources supported methodological framing, definitional context, or multiple domains simultaneously. Counts reflect primary thematic assignment, although many studies contributed to multiple conceptual mapping categories.

This integration followed three steps. First, each of the 123 included articles was assigned to one or more conceptual mapping categories based on its primary analytical focus, as defined in Table S2. Second, categories addressing related mechanisms were grouped into overarching thematic sections—for example, GABAergic, glutamatergic, and monoaminergic domains were synthesized jointly in Section 3, while state modifier domains were organized into Section 4. Third, thematic sections were sequenced to reflect a logical progression from molecular mechanisms through state-dependent modulation to clinical and translational implications. Where evidence from multiple domains converged on the same question, it was synthesized within the most conceptually relevant section.

The literature identification process is summarized in Figure S1, representative search strategies are provided in Table S1, and the data extraction fields and conceptual mapping domains are presented in Table S2.

## 3. Neurotransmitter Systems and Network Excitability

Network excitability in epilepsy emerges from the interaction of multiple neurotransmitter systems that regulate synaptic transmission and network dynamics. Among these, γ-aminobutyric acid (GABA) and glutamate represent the principal inhibitory and excitatory neurotransmitters, respectively, and have been extensively implicated in seizure generation [2,3,20,21,22,23].

Alterations in GABAergic inhibition may occur at several levels, including reduced interneuron function, impaired receptor activity, and disrupted chloride homeostasis. In particular, dysfunction of parvalbumin-positive interneurons—key regulators of fast inhibitory control and network synchronization—has been consistently reported in both experimental models and human epilepsy [6,24,25]. In parallel, changes in chloride transport mechanisms, such as altered expression of KCC2 and NKCC1 transporters, can shift GABAergic signaling toward depolarizing effects, further contributing to network hyperexcitability [25,26,27,28,29].

Glutamatergic transmission also plays a central role in seizure dynamics. Increased glutamate release, altered receptor composition (including NMDA and AMPA receptors), and impaired clearance mechanisms have all been associated with enhanced excitatory drive and seizure propagation [22,30,31,32,33,34]. Importantly, excitatory and inhibitory systems do not operate independently but are tightly coupled within dynamic networks, where even subtle shifts in their balance may lead to large-scale changes in network behavior.

Beyond classical excitatory–inhibitory interactions, additional neuromodulatory systems—including cholinergic, serotonergic, and noradrenergic pathways—contribute to the regulation of cortical excitability and seizure susceptibility [35,36,37,38,39,40,41,42]. These systems are closely linked to brain states such as arousal, attention, and stress, suggesting that neurotransmitter function cannot be fully understood without considering its broader physiological context.

At the network level, these molecular and synaptic processes converge to shape patterns of neuronal synchronization and oscillatory activity. Epileptic seizures are increasingly viewed as emergent phenomena arising from large-scale network dynamics rather than purely local abnormalities [43,44,45,46,47,48,49,50]. Disruptions in oscillatory activity, particularly in the gamma frequency range, have been associated with impaired inhibitory control and altered coordination of neuronal ensembles [8,41,42,43,44,45,46,47,48,49,50,51,52,53,54,55].

**Figure 2:**
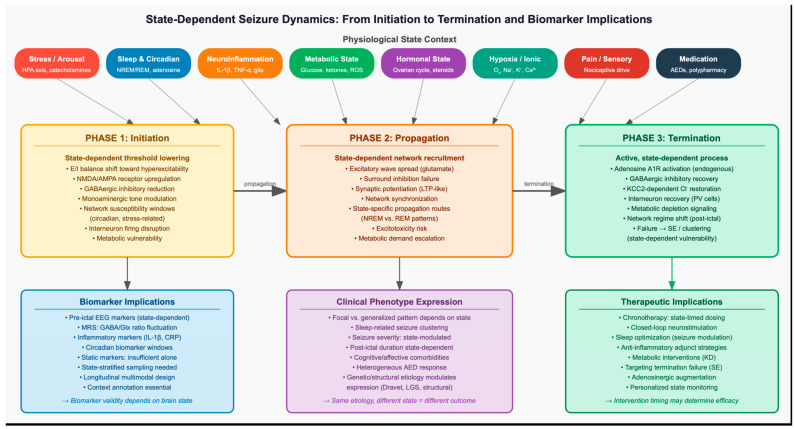
Sequential model of state-dependent seizure dynamics, illustrating the mechanistic and clinical implications of three phases: initiation, propagation, and termination. Multiple physiological state modifiers—including stress, sleep–wake cycles, neuroinflammation, metabolic conditions, hormonal state, hypoxia, pain, and medication context—shape each phase through distinct molecular and network mechanisms. During initiation, state-dependent shifts in excitation–inhibition balance, GABAergic inhibitory reduction, and monoaminergic tone modulation lower seizure threshold. During propagation, glutamatergic recruitment, network synchronization, and surround inhibition failure facilitate ictal spread. During termination, adenosinergic signaling, KCC2-dependent chloride restoration, and interneuronal recovery actively limit seizure duration; failure of these mechanisms—amplified by unfavorable brain states—contributes to seizure clustering and status epilepticus. The lower panels illustrate downstream implications for biomarker development (state-stratified, longitudinal approaches), clinical phenotype expression (etiology- and state-dependent variability), and therapeutic strategies (chronotherapy, closed-loop neurostimulation, metabolic interventions). Arrows indicate phase transitions shaped by prevailing network context, not rigid sequential causality. Importantly, seizures themselves act as state modifiers through post-ictal inflammatory, metabolic, and sleep-related effects, creating a bidirectional feedback between seizure dynamics and brain state.

This integrative relationship between state modifiers, network mechanisms, and clinical expression is schematically summarized in Figure 2.

Taken together, these findings indicate that neurotransmitter dysregulation in epilepsy is not a fixed defect but a dynamic process operating across multiple levels of organization. The functional impact of neurotransmitter systems depends not only on their baseline properties but also on their interaction with ongoing network activity and physiological state, providing a basis for understanding variability in seizure expression [8,21,37].

Section 4 extends this analysis by focusing specifically on how external and internal state modifiers dynamically reshape these neurotransmitter systems and their network-level effects. Whereas Section 3 addresses the mechanistic properties of each system, Section 4 examines the contextual factors that determine their functional expression.

Within this state-dependent model, the proposed causal hierarchy should be interpreted as probabilistic rather than strictly linear. State modifiers, such as sleep disruption, stress, inflammation, metabolic imbalance, medication context, and developmental stage, influence molecular and synaptic mechanisms, including GABAergic inhibition, glutamatergic recruitment, chloride homeostasis, adenosinergic tone, and neuromodulatory signaling. These mechanisms then alter network excitability, synchronization, and resilience, thereby changing the probability of seizure initiation, propagation, or termination failure. The dominant pathway may differ across etiologies and brain states; therefore, the framework is intended to define testable relationships rather than a single universal causal chain [5,7,14].

## 4. State-Dependent Modulation of Seizure Susceptibility

Clinical and experimental evidence consistently demonstrates that seizure susceptibility is not constant but varies over time within the same individual. This variability is closely linked to changes in physiological and pathological brain states, suggesting that network excitability is dynamically modulated rather than fixed [44,47,56].

One of the most well-established modulators of seizure susceptibility is the sleep–wake cycle. Different stages of sleep are associated with distinct patterns of neuronal synchronization and neurotransmitter activity. Non-rapid eye movement (NREM) sleep is characterized by increased neuronal synchrony and has been associated with a higher likelihood of seizure occurrence in certain epilepsy syndromes, whereas rapid eye movement (REM) sleep, with its desynchronized activity, may exert a protective effect [15,57,58,59,60,61,62].

Stress represents another critical factor influencing seizure dynamics. Both acute and chronic stress can alter neurotransmitter systems through activation of the hypothalamic–pituitary–adrenal (HPA) axis, leading to changes in cortisol levels and downstream effects on excitatory and inhibitory signaling. These processes may lower seizure threshold and contribute to increased seizure frequency in susceptible individuals [14,63,64,65].

Inflammatory processes have also emerged as important modulators of network excitability. Pro-inflammatory cytokines, including interleukin-1β and tumor necrosis factor-α, can influence synaptic transmission, receptor expression, and ion channel function, thereby promoting a pro-excitatory state within neural networks [11,66,67,68,69,70,71]. Evidence from both animal models and human studies suggests that neuroinflammation may play a role not only in epileptogenesis but also in short-term fluctuations of seizure susceptibility.

Metabolic factors further contribute to state-dependent variability. Changes in glucose availability, mitochondrial function, and energy metabolism can directly affect neuronal firing properties and synaptic efficiency. The clinical efficacy of metabolic interventions, such as ketogenic diets, underscores the importance of metabolic state in modulating seizure activity [17,72,73,74,75,76,77].

Importantly, these modulatory influences do not act independently but interact within a complex, multi-level system. Sleep, stress, inflammation, and metabolism are interconnected processes that collectively shape brain state and, consequently, network excitability. This integrated perspective suggests that seizure occurrence reflects the transient configuration of multiple interacting systems rather than the expression of a single underlying abnormality [10,15,24].

From this viewpoint, neurotransmitter systems function as mediators of state-dependent modulation, translating systemic and physiological influences into changes in synaptic and network activity. This model provides a basis for understanding why seizure risk fluctuates over time and why identical structural or molecular abnormalities may result in variable clinical manifestations [11,17,43].

An integrative summary of state-dependent modulation across neurotransmitter systems is provided in Table 2.

## 5. State-Dependent Mechanisms of Seizure Termination

Seizure termination is not a passive event but an active process dependent on the capacity of neural networks to restore inhibitory control and interrupt pathological synchronization [69]. Accumulating evidence indicates that the mechanisms governing seizure termination are as dynamically regulated as those involved in initiation, and that their efficacy is critically shaped by the prevailing brain state.

One of the most well-characterized endogenous termination mechanisms involves adenosine, a neuromodulator released in an activity-dependent manner during ictal discharges. Adenosine acts primarily through A1 receptors to suppress neuronal firing, reduce glutamate release, and enhance GABAergic tone, collectively promoting network stabilization [74,75,78]. Crucially, the availability and receptor sensitivity of the adenosine system are influenced by metabolic state and prior neuronal activity. Conditions associated with mitochondrial stress, impaired energy metabolism, or prolonged wakefulness may reduce adenosinergic efficacy, thereby compromising the network’s intrinsic capacity for self-termination [56,79,80]. This provides a mechanistic link between metabolic brain state and the duration and severity of individual seizures.

A second major mechanism involves state-dependent failure of GABAergic inhibition during sustained ictal activity. Prolonged seizures are associated with a progressive shift in chloride homeostasis, driven by activity-dependent downregulation or internalization of the potassium-chloride cotransporter KCC2 [25,29]. As intraneuronal chloride accumulates, GABAergic signaling transitions from inhibitory to depolarizing, effectively converting an inhibitory brake into an excitatory drive. This chloride collapse is not uniform but depends on prior network conditions: neuroinflammatory states, metabolic stress, and sleep deprivation have each been associated with KCC2 dysfunction, suggesting that susceptibility to inhibitory failure during seizures is itself state-dependent [11,56,81].

Interneuronal activity also plays a central role in termination dynamics. Parvalbumin-positive interneurons, which provide powerful perisomatic inhibition and regulate network synchrony, are particularly vulnerable to metabolic exhaustion and inflammatory signaling [24,56,82]. Their firing capacity during prolonged ictal discharges may be compromised under conditions of energy deficit or cytokine-mediated modulation, reducing the inhibitory recruitment necessary to interrupt pathological activity [11,56,69].

At the network level, termination requires a transition from hypersynchronous ictal dynamics to a post-ictal state characterized by reduced excitability and reorganized connectivity. This transition reflects an emergent shift in network regime rather than the simple cessation of excitatory input [47,71,83,84,85,86]. State-dependent factors, including the degree of prior synaptic potentiation, the integrity of inhibitory circuits, and the availability of neuromodulatory resources, collectively determine whether networks can achieve this transition rapidly or remain in prolonged ictal or post-ictal states.

These observations suggest that seizure duration and termination failure are not determined solely by the initiating lesion or genetic substrate but are shaped by the dynamic state of the network at the time of the event [47,70,83]. Within a state-dependent framework, conditions such as sleep deprivation, acute inflammation, or metabolic stress may not only lower seizure threshold but also impair the mechanisms that normally limit seizure duration, thereby increasing the risk of prolonged or clustered events. This has direct implications for the interpretation of seizure variability and for identifying state-sensitive targets for therapeutic intervention.

Experimentally, initiation and termination mechanisms may be partially dissociated by focusing on distinct outcome measures [69,87]. Initiation-related mechanisms can be assessed through seizure threshold, latency to first seizure, or probability of ictal onset under controlled perturbations. Termination-related mechanisms, by contrast, require analysis of seizure duration, probability of spontaneous self-termination, transition into post-ictal suppression, seizure clustering, and progression toward status epilepticus. This distinction is important because an intervention may raise seizure threshold without necessarily improving termination capacity or conversely may shorten seizure duration without preventing seizure onset. State-dependent analysis of termination therefore requires designs that measure not only whether seizures occur, but also how long they persist and how efficiently networks return to a non-ictal regime. These mechanisms may be particularly relevant in refractory epilepsies characterized by prolonged seizures or frequent seizure clusters, such as Lennox–Gastaut syndrome. In such conditions, termination failure may reflect not only high baseline epileptogenicity but also impaired state-dependent recovery mechanisms, including reduced inhibitory reserve, altered chloride regulation, metabolic exhaustion, and insufficient neuromodulatory stabilization. However, because these syndromes are etiologically heterogeneous, the proposed mechanisms should be interpreted as convergent downstream processes rather than syndrome-specific explanations.

It is also important to recognize that seizures themselves function as state modifiers. Recurrent ictal events induce post-ictal neuroinflammation, metabolic exhaustion, disruption of sleep architecture, and transient alterations in chloride homeostasis, all of which influence subsequent seizure susceptibility and termination capacity. This bidirectional relationship—in which brain state modulates seizure dynamics and seizures in turn reshape brain state—represents a self-reinforcing cycle that may contribute to seizure clustering and disease progression in susceptible individuals. Accounting for this feedback is essential for interpreting the temporal dynamics of seizure occurrence and for designing state-informed therapeutic strategies [10,38,56,69].

## 6. Implications for Biomarker Development

The identification of reliable biomarkers in epilepsy remains a major challenge, particularly in the context of predicting seizure occurrence and treatment response. Despite extensive research efforts, most currently available biomarkers are based on static measurements, including interictal electrophysiological patterns, neuroimaging findings, or baseline molecular profiles [9,88,89,90,91].

However, the clinical variability of epilepsy suggests that such static approaches may be insufficient. Seizure occurrence is inherently dynamic, and risk fluctuates over time depending on the underlying brain state. As a result, biomarkers that do not account for temporal variability may fail to capture clinically relevant changes in network excitability [16,92,93].

Electrophysiological markers, such as interictal epileptiform discharges and spectral features of EEG activity, provide important information about network dynamics but are often limited by their context dependence. Their predictive value may vary depending on factors such as sleep stage, recent seizure history, and external modulators, highlighting the need for more temporally sensitive approaches [15,46,52,91,94].

Similarly, molecular and biochemical markers—including inflammatory mediators, neurotransmitter levels, and metabolic indicators—have shown associations with seizure activity, but their interpretation is complicated by substantial intra-individual variability and sensitivity to physiological conditions [11,56,71,95]. These findings suggest that biomarker expression is not fixed but reflects ongoing interactions between molecular processes and brain state.

Neuroimaging biomarkers, while providing valuable structural and functional information, also face limitations in capturing dynamic changes in network excitability. Most imaging techniques offer snapshots of brain organization rather than continuous measures of functional variability, which may reduce their utility in predicting short-term seizure risk [88,96,97,98].

Within a state-dependent framework, biomarkers can be conceptualized not as stable traits but as dynamic indicators of network configuration. This perspective emphasizes the importance of longitudinal and multimodal approaches, integrating electrophysiological, molecular, and behavioral data over time [98,99,100,101,102,103,104,105,106].

Clinically feasible state-sensitive biomarkers may include several categories. First, electrophysiological markers, such as changes in interictal epileptiform discharge burden, spectral power, phase–amplitude coupling, or sleep-stage–dependent EEG features, may provide temporally sensitive indicators of network excitability. Second, autonomic and behavioral markers—including heart-rate variability (HRV), electrodermal activity, actigraphy-derived sleep metrics, and patient-reported digital diary records of seizure occurrence, stress, and sleep quality—may serve as accessible proxies of ongoing brain state. Third, biochemical markers—including inflammatory mediators such as interleukin-1β (IL-1β), tumor necrosis factor-α (TNF-α), and C-reactive protein, as well as metabolic indicators such as blood glucose, lactate levels, and indices of ketone body availability—may be informative when interpreted longitudinally and in relation to clinical context rather than as isolated measurements. Fourth, treatment-related markers, including changes in seizure clustering after sleep deprivation, medication timing, or metabolic intervention, may provide indirect evidence of state-dependent modulation.

For clinical interpretation, it is useful to distinguish markers of general seizure susceptibility from markers with potential pre-ictal predictive value. General susceptibility markers may include structural lesions, baseline interictal epileptiform activity, or chronic inflammatory/metabolic profiles. By contrast, candidate pre-ictal markers should demonstrate temporal proximity to seizure onset, typically within minutes to hours before seizure onset, and reproducible change within an individual before seizures occur.

Potential pre-ictal indicators include dynamic EEG features such as changes in spectral power, synchronization, phase–amplitude coupling, or evolving interictal discharge burden; autonomic markers such as heart-rate variability; sleep-stage transitions or sleep fragmentation; and time-stamped behavioral or digital diary signals. At present, the most consistent evidence—supported by multiple prospective ambulatory EEG studies and algorithmic seizure prediction trials—is available for electrophysiological markers, particularly interictal EEG features and circadian/cyclic seizure patterns [92,93,96], whereas autonomic, biochemical, and digital behavioral markers remain more exploratory and require further prospective validation. Therefore, these markers should not be interpreted as established predictors in isolation, but as components of multimodal and longitudinal risk models.

However, these markers differ substantially in feasibility, reproducibility, and scalability. EEG-derived and wearable-derived measures are more suitable for longitudinal monitoring, whereas molecular markers may be less scalable and more sensitive to sampling conditions [89,93]. Therefore, state-sensitive biomarker development should prioritize repeated, multimodal, and time-stamped measurements rather than single-timepoint assessments.

Such an approach may improve the ability to identify periods of increased seizure susceptibility and to better understand variability in treatment response. Rather than relying on single measurements, future biomarker strategies may benefit from incorporating temporal patterns, context-specific signals, and individual variability [94,100,101].

Overall, recognizing the state-dependent nature of neurotransmitter function and network excitability provides a more coherent basis for interpreting biomarker data and may support the development of more individualized and clinically meaningful predictive models in epilepsy.

## 7. Discussion

The narrative synthesis identified several key findings. First, neurotransmitter dysregulation in epilepsy is better understood as a dynamic, state-dependent process than as a fixed abnormality. Second, physiological and pathological modifiers, including sleep, stress, inflammation, metabolic state, hormonal context, pain, infection, hypoxia, and ionic imbalance, may alter seizure susceptibility through convergent effects on molecular, synaptic, and network mechanisms. Third, seizure termination appears to represent an active state-dependent process, not merely passive cessation of ictal activity. Fourth, biomarker interpretation requires temporal and contextual stratification, because the same marker may have different predictive value across brain states.

This review is narrative in nature and does not aim to provide a systematic synthesis of all available evidence but rather to propose a conceptual approach integrating molecular and network-level perspectives.

The present review integrates evidence from molecular, cellular, and network-level studies to examine neurotransmitter dysregulation in epilepsy within a state-dependent framework. The findings discussed above suggest that seizure generation and variability cannot be fully explained by static alterations in excitatory and inhibitory balance alone but instead reflect dynamic interactions across multiple levels of brain organization [45,46,47,48,50].

One of the central implications of this perspective is the recognition that network excitability is not a fixed property but a context-dependent variable. Factors such as sleep, stress, inflammation, and metabolic state continuously modulate neurotransmitter function and network dynamics, leading to fluctuations in seizure susceptibility over time. This helps explain why patients with similar structural or genetic backgrounds may exhibit markedly different clinical trajectories and treatment responses [10,89,107,108,109,110,111,112]. The clinical burden of epilepsy extends beyond seizure frequency and includes increased mortality risk, further reinforcing the need for comprehensive monitoring and individualized management [113].

Importantly, this model does not contradict existing models of epilepsy but rather extends them. Classical concepts of excitatory–inhibitory imbalance remain relevant; however, they may be more accurately understood as components of a dynamic system whose functional expression depends on the current physiological state. In this sense, neurotransmitter dysregulation may represent a downstream manifestation of broader network instability rather than a single primary cause [12,87,114,115,116,117].

The state-dependent perspective also provides a useful context for interpreting the limitations of current biomarker approaches. As discussed, many biomarkers are derived from static measurements that do not capture temporal variability. Integrating state-related information may improve their interpretability and clinical relevance, particularly in predicting seizure risk and treatment response [88,89,93].

This perspective is also consistent with emerging approaches in state-dependent neuromodulation and chronotherapy. Recent studies have demonstrated that the efficacy of neurostimulation and pharmacological interventions may depend on the timing of application relative to endogenous brain states, including circadian rhythms, sleep stages, and stress-related fluctuations [17,118]. These findings support the idea that therapeutic interventions may benefit from being aligned with state-dependent variations in network excitability rather than applied uniformly across time. While such approaches remain largely experimental, they provide a potential translational extension of the framework proposed in this review.

### 7.1. Quantitative Translation and Clinical Implications

The state-dependent model proposed here generates several empirically testable predictions. Seizure susceptibility is expected to show systematic variation across physiological states—including sleep stages, circadian phase, stress exposure, and metabolic context—and this variation should be detectable through repeated electrophysiological or physiological measurement. State-stratified longitudinal EEG studies, within-individual comparison of biomarker performance across different physiological conditions, and prospective ambulatory monitoring designs represent feasible approaches for validating or refuting these predictions. Specifically, if the proposed model is correct, biomarkers sampled during defined physiological states (e.g., NREM sleep, post-stress periods, or metabolically compromised states) should demonstrate stronger and more reproducible associations with subsequent seizure occurrence than biomarkers obtained without state annotation.

In practical terms, the state-dependent interpretation of seizure susceptibility proposed in this review may influence clinical decision-making by encouraging clinicians to interpret seizure variability in relation to modifiable state factors rather than only baseline epilepsy type. For example, recurrent seizure clustering after sleep loss may support prioritization of sleep stabilization and circadian regularity; stress-linked exacerbations may justify structured stress-management interventions as adjunctive care; and seizures occurring in relation to metabolic disruption, medication timing, or polypharmacy may prompt reassessment of dosing schedules and systemic contributors. These examples are not intended to replace standard antiseizure treatment, but to illustrate how state-aware assessment may complement conventional management [24,27].

From a conceptual standpoint, the findings reviewed here support the idea that epilepsy can be understood as a disorder of dynamic network instability, in which transient configurations of neural activity determine the emergence of pathological events. Within this view, different clinical conditions may share partially overlapping mechanisms related to impaired regulation of excitability, even when their etiologies differ. However, the degree of overlap and its clinical significance require careful evaluation and should not be overgeneralized [8,63,67].

The applicability of the state-dependent neurotransmitter model proposed in this review is therefore not uniform across all epilepsies. It is likely to be most informative in contexts where seizure expression fluctuates substantially over time and where physiological state modifiers, such as sleep, stress, inflammation, metabolic status, medication exposure, or hormonal state, plausibly modulate network excitability. By contrast, its explanatory value may be more limited in conditions dominated by a fixed structural substrate, rapidly progressive destructive pathology, severe developmental disruption, or highly penetrant monogenic mechanisms in which upstream causation strongly constrains downstream network behavior. In such cases, the state-dependent framework should be regarded as complementary rather than primary.

Neuropsychiatric comorbidities, particularly depression and anxiety, represent a substantial source of disease burden in epilepsy and may reflect partially overlapping neurobiological vulnerabilities [21,28,107,119,120,121,122].

These considerations are consistent with emerging efforts to develop integrative approaches that link molecular mechanisms with large-scale network behavior. Such approaches emphasize the importance of multi-level analysis and may facilitate the development of more precise and individualized therapeutic strategies [95,108]. At the same time, it is important to acknowledge that current evidence remains heterogeneous, and many of the proposed mechanisms are supported primarily by experimental or indirect clinical data. Drug resistance affects a substantial proportion of patients with epilepsy and remains a major unresolved challenge for individualized treatment [112,122,123].

Several limitations should be considered when interpreting this review. First, as a narrative synthesis, the selection of studies was not based on formal systematic criteria, which may introduce selection bias. Second, the available evidence varies in methodological quality, and not all findings are directly comparable across studies. The absence of formal quality appraisal procedures means that studies of varying methodological rigor were treated as broadly equivalent within the conceptual synthesis, which may affect the robustness of some conclusions. Methodological heterogeneity and concerns about statistical power further complicate cross-study interpretation [123,124,125,126]. In addition, the applicability of this model to pediatric and developmental epilepsies may vary, since chloride regulation, inhibitory maturation, and network organization are strongly age-dependent. Third, the concept of state-dependent modulation, while supported by multiple lines of evidence, still requires further validation in prospective and longitudinal studies.

From a clinical perspective, the state-dependent model proposed here suggests that treatment strategies may benefit from considering temporal fluctuations in brain state. For example, optimization of sleep patterns, stress management, and metabolic regulation may influence seizure susceptibility beyond baseline pharmacological effects. In addition, future approaches such as closed-loop neurostimulation or adaptive pharmacotherapy may incorporate state-dependent signals to improve therapeutic precision [105,127]. However, it should be emphasized that most of these approaches remain exploratory and require further validation in controlled clinical studies.

Despite these limitations, the state-dependent framework offers a structured approach to integrating diverse findings and to better understanding variability in epilepsy. By focusing on dynamic interactions rather than static abnormalities, this perspective may help bridge the gap between molecular mechanisms, network behavior, and clinical expression. Critically, the present model draws attention to seizure termination as an underexplored but clinically relevant dimension of seizure dynamics—one that is shaped by the same state-dependent factors that influence initiation and propagation. A more complete account of seizure biology requires not only understanding why seizures begin, but why they sometimes fail to stop. Incorporating termination mechanisms into models of neurotransmitter dysregulation and network excitability may therefore open new directions for both biomarker research and the development of context-sensitive therapeutic interventions.

### 7.2. Testable Predictions and Future Research Directions

Several testable predictions follow from the state-dependent model proposed here. First, the predictive value of electrophysiological biomarkers should vary across brain states; for example, EEG features obtained during sleep deprivation or specific sleep stages may differ in prognostic value from those recorded during wakeful baseline conditions. Second, intra-individual changes in seizure susceptibility should correlate more strongly with longitudinal state variables, such as sleep quality, circadian phase, stress exposure, metabolic status, or inflammatory activity, than with single-timepoint measurements alone. Third, termination-related outcomes, including seizure duration, clustering, and post-ictal recovery, should be modulated by the same state variables that influence initiation, but not necessarily in the same direction. Fourth, state-stratified biomarker models should outperform static models in predicting seizure risk or treatment response. Fifth, interventions timed according to state variables, such as chronotherapy, closed-loop neurostimulation, or sleep-targeted strategies, should show greater efficacy than non-stratified approaches in selected patient subgroups.

These predictions could be tested using prospective longitudinal designs combining repeated EEG recordings, wearable-derived autonomic and sleep metrics, seizure diaries, medication timing, and selected molecular or metabolic markers. Preclinical studies could further isolate causal pathways by experimentally manipulating sleep deprivation, inflammatory activation, metabolic stress, or neuromodulatory tone while separately measuring seizure threshold, propagation, and termination outcomes.

## 8. Conclusions

This review highlights that neurotransmitter dysregulation in epilepsy is better understood as a dynamic, state-dependent process rather than a fixed abnormality. Evidence from molecular, cellular, and network-level studies indicates that network excitability is continuously modulated by physiological and pathological factors, including sleep–wake cycles, stress, neuroinflammation, and metabolic conditions, as well as pain, infection, hypoxia, and ionic/electrolyte imbalance. These factors do not merely trigger seizures from a stable baseline but actively reshape the molecular and network conditions that determine whether seizures initiate, propagate, and terminate.

A central contribution of this framework is the recognition that seizure termination, like initiation, is a state-dependent process. The capacity of neural networks to restore inhibitory control and interrupt ictal activity depends on the integrity of adenosinergic signaling, chloride homeostasis, and interneuronal function—all of which are sensitive to the prevailing brain state. Conditions such as sleep deprivation, metabolic stress, or neuroinflammation may therefore not only lower seizure threshold but also impair endogenous termination mechanisms, contributing to prolonged or clustered seizure events.

Experimentally, initiation and termination mechanisms can be dissociated through distinct outcome measures: seizure threshold and latency index the susceptibility to initiation, whereas seizure duration, self-termination probability, and susceptibility to clustering or status epilepticus reflect the integrity of active termination processes. This distinction is clinically and experimentally relevant, as interventions may differentially target one phase without necessarily affecting the other.

Recognizing the state-dependent nature of these mechanisms provides a more coherent explanation for the variability of seizure occurrence, duration, and treatment response observed in clinical practice. It also underscores the limitations of static approaches to biomarker development. Single-timepoint measurements of neurotransmitter markers, electrophysiological features, or inflammatory indicators may reflect the current network state rather than stable disease traits, limiting their predictive value when applied without contextual information. Longitudinal and multimodal approaches that incorporate state-related variables—including sleep quality, stress exposure, and metabolic indicators—may improve biomarker interpretability and support more individualized seizure risk assessment.

This model does not replace existing models of epilepsy but extends them by integrating multiple levels of organization and emphasizing their dynamic interactions. Future research should prioritize longitudinal designs with repeated state-stratified assessments, combining electrophysiological monitoring with molecular and behavioral measures. Particular attention should be given to the temporal relationship between state transitions and changes in seizure susceptibility and termination capacity. Such investigations may facilitate the identification of time-sensitive biomarker windows and support the development of state-aware, individualized therapeutic strategies in epilepsy.

## Figures and Tables

**Table 1:** Etiological heterogeneity of epilepsy and its interpretation within a state-dependent neurotransmitter model. The table summarizes major etiological categories and illustrates how diverse upstream causes may converge on shared downstream mechanisms of network instability and altered neurotransmitter regulation.

Etiological Category	Primary Level of Pathology	Examples of Mechanistic Emphasis	Why a Downstream Neurotransmitter/Network Framework May Still Be Relevant
Genetic epilepsies/monogenic channelopathies, including genetic developmental and epileptic encephalopathies Examples include SCN1A-related epilepsies, KCNQ2- and SCN2A-related epilepsies, and other channelopathies. (Note: Dravet syndrome is a genetically defined developmental and epileptic encephalopathy associated most commonly with SCN1A pathogenic variants and is therefore discussed under the DEE row below.)	Ion channels, receptors, synaptic proteins	SCN-related channel dysfunction, receptor abnormalities, altered synaptic signaling	Helps interpret how inherited molecular abnormalities are translated into state-dependent excitability and phenotypic variability
Structural epilepsies	Lesion-associated circuit reorganization	Hippocampal sclerosis, cortical dysplasia, mTOR-related lesional signaling, post-injury reorganization	Useful for understanding how lesion-driven networks express variable seizure thresholds, mood symptoms, and cognitive dysfunction
Developmental and epileptic encephalopathies with heterogeneous etiologies Examples include West syndrome, Lennox–Gastaut syndrome, and other age-dependent epileptic encephalopathies.	Circuit maturation and developmental network formation	Age-dependent synaptic and connectivity disturbances, altered chloride homeostasis, developmental circuit instability	Important for interpreting age-sensitive state modulation and developmental differences in phenotype
Metabolic/immune/other systemic epilepsies (Rasmussen syndrome; immune-mediated epilepsies)	Bioenergetic, inflammatory, or multisystem processes	Metabolic stress, immune signaling, neurometabolic instability	Supports integration of metabolism, inflammation, and state-dependent vulnerability
Unknown/mixed etiologies	Multifactorial or unresolved mechanisms	Combined network, molecular, and environmental contributions	Highlights the value of downstream interpretive models when upstream causation remains uncertain

**Table 2:** State-dependent modulation of neurotransmitter systems in epilepsy. The table summarizes how physiological and pathological states (e.g., sleep, stress, inflammation, metabolic factors) influence neurotransmitter function, network excitability, and seizure susceptibility.

State Modifier	GABAergic Signaling	Glutamatergic Signaling	Monoaminergic Modulation	Network and Clinical Expression
**Stress/Arousal (HPA axis activation)**	Reduced phasic inhibition; altered GABA_A receptor trafficking; glucocorticoid-mediated modulation	Enhanced NMDA receptor activity; increased calcium influx; stress-facilitated plasticity	Elevated noradrenergic tone; context-dependent serotonergic shifts	Hyperarousal-biased network states; reduced inhibitory precision; increased seizure susceptibility; anxiety and mood instability
**Sleep and circadian disruption (sleep deprivation; fragmented sleep; circadian misalignment)**	Reduced tonic inhibition; altered interneuron firing patterns	Impaired synaptic downscaling; altered plasticity recalibration	Circadian fluctuations in serotonergic and noradrenergic tone	Increased network noise; reduced resilience; seizure clustering; cognitive impairment; mood disturbances
**Neuroinflammatory state (microglial activation; cytokines IL-1β, TNF-α)**	Impaired astrocytic GABA uptake; cytokine-mediated receptor modulation	Reduced glutamate clearance; increased excitotoxic vulnerability	Altered monoamine synthesis and receptor sensitivity	Reduced network stability; maladaptive plasticity; seizures; depression; anxiety; cognitive decline
**Metabolic state (glucose vs. ketone metabolism; mitochondrial efficiency)**	Metabolism-dependent modulation of inhibitory tone	Energy-sensitive regulation of excitatory transmission	Redox- and metabolism-sensitive neuromodulatory tone	Shifts in excitability thresholds; altered oscillatory coordination; seizure modulation; fatigue; cognitive variability
**Medication/polypharmacy context (AEDs; psychotropic drugs)**	Pharmacological enhancement of inhibition; tolerance and adaptive changes	Indirect modulation; interference with plasticity mechanisms	Drug–drug interactions affecting neuromodulatory tone	Context-dependent stabilization or sedation; variable seizure control; cognitive slowing; mood changes
**Hormonal/ionic/hypoxic state**	Fluctuating GABAergic tone; altered chloride homeostasis	Changes in glutamatergic excitability under ionic or hypoxic stress	Context-dependent neuromodulatory shifts	Catamenial or hormonally linked seizure vulnerability; increased excitability during electrolyte imbalance or hypoxia
**Pain/infection/systemic stress**	Inflammation-associated impairment of inhibitory control	Cytokine- and stress-mediated facilitation of excitatory signaling	Arousal- and sickness-related monoaminergic changes	Transient lowering of seizure threshold; seizure clustering during systemic illness or pain-related stress

Abbreviations: AEDs, antiepileptic drugs; GABA, γ-aminobutyric acid; GABA_A, ionotropic γ-aminobutyric acid type A receptor; HPA axis, hypothalamic–pituitary–adrenal axis; IL-1β, interleukin-1 beta; NMDA, N-methyl-D-aspartate; TNF-α, tumor necrosis factor alpha.

## Data Availability

No new data were created or analyzed in this study. Data sharing is not applicable to this article.

## Supplementary Materials

The following supporting information can be downloaded at: https://www.mdpi.com/article/10.3390/biology15110850/s1, Figure S1: Literature identification and study selection flow diagram; Table S1: Representative database search strategies; Table S2: Data extraction fields and conceptual mapping domains.
