# Supplementary material for: State-Dependent Modulation of Neurotransmitter Systems in Epilepsy: A Mechanistic Framework for Seizure Dynamics and Biomarker Variability
Source: Biology (Basel). 2026 May 29;15(11):850. doi: 10.3390/biology15110850 (PMC13256034; doi:10.3390/biology15110850)
Supplement: Supplementary file 1 [file biology-15-00850-s001.zip › biology-4299614-supplementary.pdf]

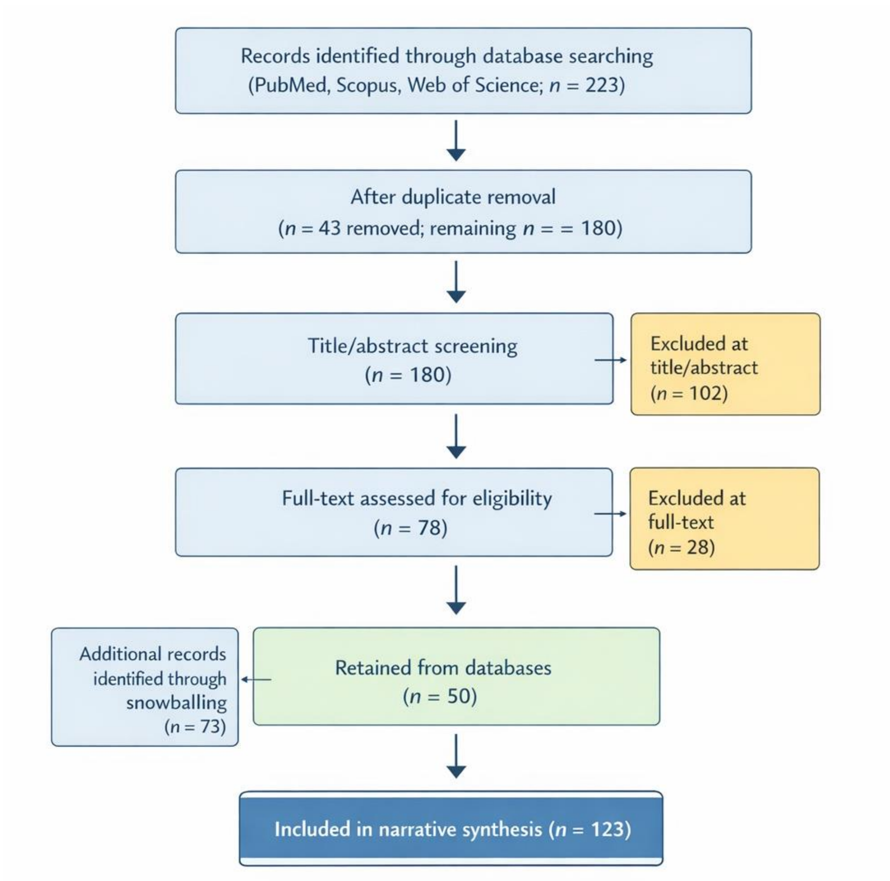

Figure S1. Literature identification and study selection flow diagram.

Table S1. Representative search strategies used across electronic databases for literature identification.

Database	Date searched	Time span filter	Language filter	Full search string	Records retrieved (n)
PubMed / MEDLINE	Nov 2025 –Jan 2026	1995–2026	English	("epilepsy" OR "seizure") AND (GABA OR glutamate OR serotonin OR monoamine*) AND (stress OR arousal OR sleep OR circadian OR inflammation OR metabolic OR medication)	n = 84
Scopus	Nov 2025 –Jan 2026	1995–2026	English	TITLE-ABS-KEY (epilepsy OR seizure*) AND (GABA OR glutamate OR monoamine* OR serotonin) AND (stress OR sleep OR circadian OR inflammation OR metabolism OR polypharmacy)	n = 65
Web of Science	Nov 2025 –Jan 2026	1995–2026	English	TS = (epilepsy OR seizure*) AND TS = (GABA OR glutamate OR monoamine* OR serotonin) AND TS = (stress OR sleep OR circadian OR inflammation OR metabolic OR medication)	n = 74

Note: Search strings shown are representative of the queries used. Minor variations in field tags and operator syntax were applied across databases to accommodate platform-specific requirements. Total combined yield before duplicate removal: n = 223

Table S2. Data extraction fields and conceptual mapping domains used to organize the narrative synthesis.

Domain	Description
Study type	Experimental animal study, human observational study, neurophysiological study, or mechanistic review
Neurotransmitter system(s)	GABAergic, glutamatergic, monoaminergic (serotonergic, noradrenergic, dopaminergic), or combinations
State modifier(s)	Stress/arousal, sleep/circadian disruption, neuroinflammation, metabolic state, medication/polypharmacy, hormonal state, pain, infection/systemic illness, hypoxia, ionic/electrolyte imbalance, and developmental stage
Level of analysis	Molecular (receptors, transporters, cytokines), synaptic (plasticity, inhibition/excitation), network (connectivity, oscillations, regime switching)
Main reported association	Seizure susceptibility, seizure duration and termination, biomarker variability, cognitive impairment, sleep-related effects
Key mechanistic link	Brief description of the proposed mechanism (e.g., altered E/I balance, impaired plasticity, reduced network stability)
Notes for framework integration	How the findings inform the state-dependent network model, seizure dynamics, termination mechanisms, and biomarker variability
